# Dietary Influences on Skin Health in Common Dermatological Disorders

**DOI:** 10.7759/cureus.55282

**Published:** 2024-02-29

**Authors:** Nidhi Sharma, Sachin M Chaudhary, Niharika Khungar, Smriti K Aulakh, Hadeeqa Idris, Ajay Singh, Kriti Sharma

**Affiliations:** 1 Dermatology, Dermdoctors Clinic, Amritsar, IND; 2 Internal Medicine, Gujarat Cancer Society (GCS) Medical College, Hospital and Research Centre, Ahmedabad, IND; 3 Internal Medicine, Sri Guru Ramdas University of Health Science and Research, Amritsar, IND; 4 Internal Medicine, Shifa International Hospital, Islamabad, PAK; 5 Internal Medicine, Sri Ram Murti Smarak Institute of Medical Sciences, Bareilly, IND; 6 Internal Medicine, Government Medical College, Amritsar, Amritsar, IND

**Keywords:** atopic dermatitis, psoriasis, immune-mediated skin diseases, diet, nutrition

## Abstract

The role of diet in the development of skin disorders is well-established, with nutritional deficiency often identified as a risk factor for skin diseases. Imbalances in the skin can be caused by nutritional deficiencies, excessive intake, insufficient nutrients, and hazardous ingredients. Patients frequently inquire about the impact of dietary patterns on skin health when consulting dermatologists in clinical settings. Simultaneously, the popularity of using nutritional supplements containing vitamins, minerals, and nutraceutical blends has been on the rise. It is crucial for dermatologists, primary care physicians, and other healthcare providers to be acquainted with evidence-based dietary interventions, distinguishing them from those that are more market-driven than truly efficacious. This review explores the modification of diet, encompassing both dietary exclusion and supplementation, as a therapeutic approach for conditions such as psoriasis, atopic dermatitis, bullous disease, vitiligo, and alopecia areata. A comprehensive literature search, utilizing the PubMed/Medline, Google Scholar, and Medscape databases, was conducted to investigate the relationship between each nutrient and various inflammatory skin diseases. The findings emphasize the significance of a well-balanced and thoughtfully planned diet in supplying adequate amounts of proteins, vitamins, and minerals to support optimal skin health. Additionally, this comprehensive review navigates through various dietary recommendations, offering insights into their multifaceted impacts on the immune system, gut microbiome, and skin health. The goal is to pave the way for informed and targeted dietary interventions for individuals dealing with food allergies and associated skin conditions.

## Introduction and background

Nutrition plays a crucial role in various biological processes affecting skin health, aging, and disease. The skin's ability to heal and resist damage is closely linked to dietary habits and nutritional conditions. Recent research has highlighted the association between health, nutrition, and eating behaviors with skin health. Clinical studies have successfully connected nutrition with tissue and organ health, emphasizing the moderate impact of nutritional status on skin health and aging. Dietary imbalances, deficiencies, excesses, and hazardous ingredients can disrupt the balance of the skin, leading to visible signs associated with vitamin, mineral, and fatty acid deficits. Emerging research suggests that dietary changes can be part of the treatment plan for various skin conditions, ranging from eczema and psoriasis to alopecia and vitiligo [1].

Although glucose is the primary fuel for skin cells, its abnormal management can significantly impact skin structure and appearance [2]. Specific nutrients such as folic acid and protein become crucial in cases of excessive skin inflammation [1]. The effects of many micronutrients on skin health are well-understood, but the impact of micronutrient supplementation on skin health is still an area of active research [3].

The objective of this study is to provide dermatologists, healthcare professionals, and patients with reliable and current information to guide dietary recommendations for enhancing skin health and managing skin illnesses. This review explores the direct and indirect pathways through which diet influences dermatological disorders, including its effects on the immune system, gut microbiota, hormone balance, oxidative stress, and inflammation.

## Review

Nutritional implications in dermatological disorders

Psoriasis

**Figure 1:**
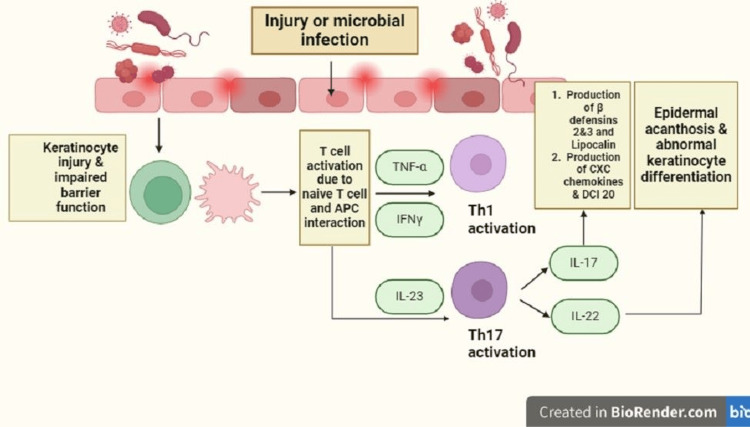
Pathogenesis of psoriasis Exposure to microbial or mechanical injury-induced damage leads to the activation of antigen-presenting cells (APCs), such as macrophages and dermal dendritic cells. The failure to maintain the skin barrier, often due to the deletion of late cornified envelope proteins 3C/3B, results in continuous exposure to these antigens. The interaction between APCs and T cells triggers the activation of Th1 and Th17 cells, mediated by IL-23. Subsequently, Th17 cells release IL-17 and IL-22, while Th1 cells produce tumor necrosis factor-alpha (TNF-α) and interferon-gamma (IFN-γ), perpetuating keratinocyte injury. This establishes a vicious positive feedback cycle exacerbating skin damage.

Psoriasis, affecting 2-3% of the population, is a chronic inflammatory condition influenced by genetic and environmental factors (Figure 1). The gut-skin axis plays a crucial role in psoriasis pathogenesis by linking gut microbiota imbalance to skin inflammation. Dysbiosis in the gut can trigger immune responses, leading to systemic inflammation and exacerbating psoriatic symptoms, highlighting the interconnectedness of gut health and skin conditions such as psoriasis [4]. The National Psoriasis Foundation recommended a hypocaloric diet in 2018, showing evidence of reduced severity and improved quality of life. A Mediterranean diet and gluten-free diet (GFD) also demonstrate potential benefits [5]. The Mediterranean diet emphasizes plant-based foods, such as fruits, vegetables, whole grains, nuts, and legumes. Psoriasis severity is exacerbated by sugars, red meat, and alcohol, activating inflammatory pathways, while nutrients such as n-3 polyunsaturated fatty acids and vitamins D and B12 mitigate inflammation. There is speculation that psoriasis might be correlated with deficiencies in vitamin D and selenium, although this link has not been conclusively demonstrated [6].

Alopecia Areata (AA)

**Figure 2:**
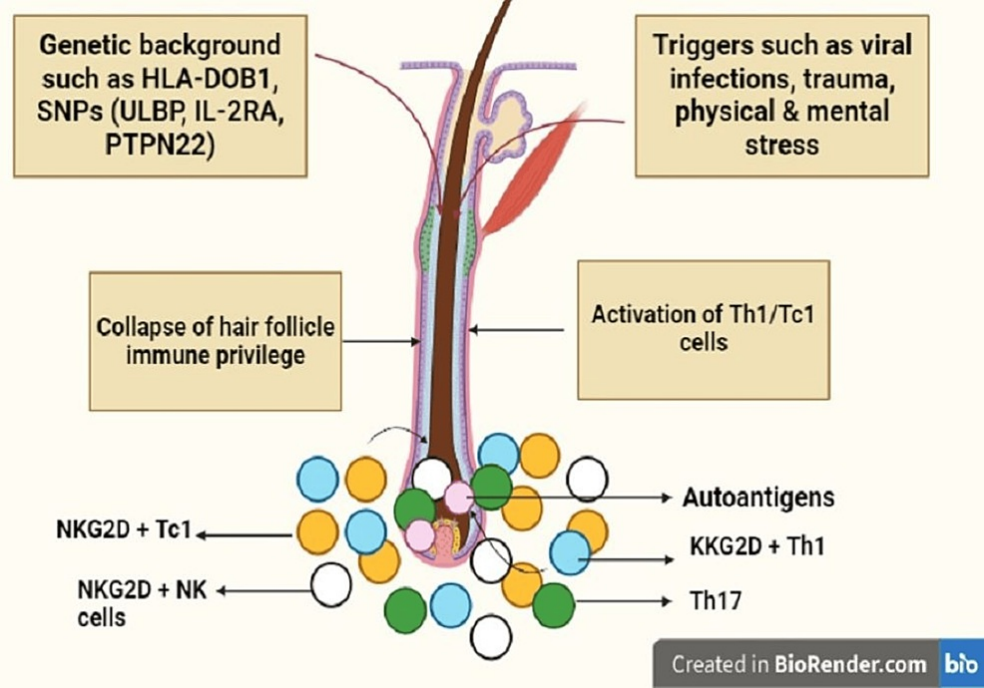
Pathogenesis of alopecia areata Within a genetic predisposition, various triggers, such as viral infections, trauma, and physical/emotional stresses activate autoreactive CD8+ T cells and Th1 cells, leading to the production of IFN-?. IFN-?, in turn, increases the expression of CXCL10 and stimulates the production of IL-15, along with its chaperone receptor IL-15Rα by hair follicle keratinocytes in the outer root sheath. Additionally, IFN-? disrupts the immune privilege of hair follicles, exposing autoantigens to autoreactive CD8+ T cells. Chemotactic activity towards CXCL10 is exhibited by Th1 and Tc1 cells, with the effector cell being NKG2D+CD8+ T cells in alopecia areata pathogenesis.

AA is a common autoimmune-driven hair loss (Figure 2), affecting around 2% of people with varying clinical symptoms, from localized patches to total body hair loss [7]. Vitamin D plays a crucial role in keratinocyte proliferation, and its deficiency has links to autoimmune disorders [8]. Topical calcipotriol, a vitamin D analog, has shown efficacy in treating AA only when used as an adjuvant. Anti-inflammatory diets, including gluten-free options and diets rich in antioxidants, are considered for their potential in reducing inflammation associated with AA [9-10]. Micronutrients such as zinc and iron play roles in the hair follicle cycle, but their therapeutic impact in oral form for AA remains inconclusive [11]. Treatment with 50,000 IU vitamin D3 for six months and three months, respectively, followed by 1,000 IU/daily as a maintenance dose, resulted in noticeable hair regrowth and resolved the deficiency state [9]. Metabolic syndrome is implicated in AA development, emphasizing the need for further research on the role of vitamin D in immunomodulation for effective treatment strategies.

Pemphigus

**Figure 3:**
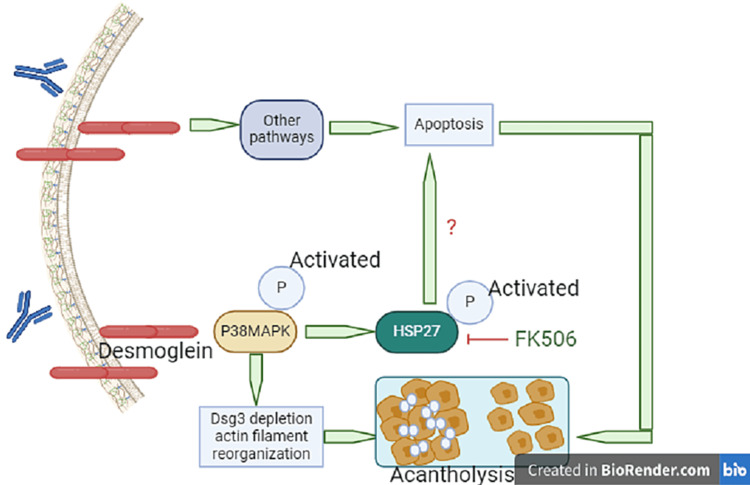
Apoptosis in the pathogenesis of pemphigus vulgaris PV-IgG depletes desmoglein (Dsg)3 and causes actin filament reorganization, leading to acantholysis. FK506 blocks heat shock protein (HSP)27 phosphorylation and presents changes in Dsg3 depletion and actin filament reorganization.

Pemphigus, a blistering disease, results from autoantibodies targeting desmoglein cadherins (Figure 3), with variants such as pemphigus vulgaris (PV), pemphigus foliaceus (PF), and severe paraneoplastic pemphigus (PNP) associated with hematologic neoplasms [11]. Traditional, but still conventional, corticosteroid therapy, while effective, poses a high fatality risk, such as aseptic joint necrosis, osteoporosis, adrenal insufficiency, gastrointestinal, hepatic, and ophthalmologic effects, hyperlipidemia, growth suppression, and possible congenital malformations. Nutritional considerations in treatment involve addressing feeding challenges, catabolism from epidermal detachment, and hydroelectrolytic imbalances [12]. Severe cases require aggressive nutritional support, advocating a high-protein diet, and, in extreme cases, a nasogastric feeding tube [13]. Vitamin D3 supplementation may have a role in pemphigus pharmacological treatment. Dietary factors, including compounds such as tannins and phenols, are implicated in pemphigus induction, with various foods and beverages identified as potential triggers, emphasizing the need for tailored nutritional management in pemphigus cases [14]. However, diet plays a probable but not definitive role in pemphigus.

Vitiligo

**Figure 4:**
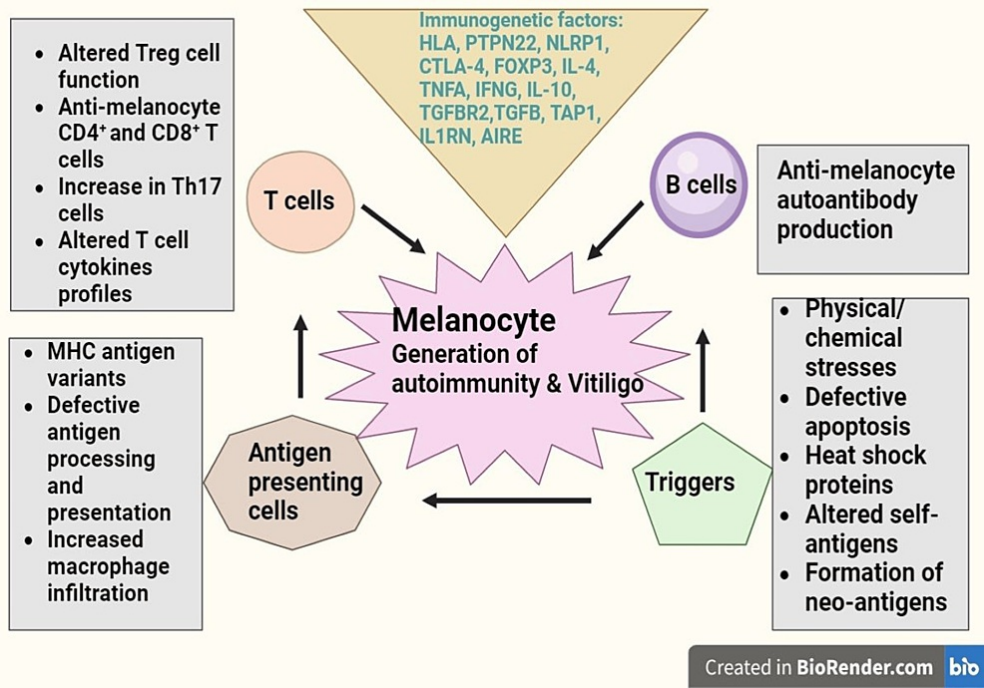
The generation of autoimmune responses against melanocytes in vitiligo Vitiligo depigmentation arises from an exaggerated immune system reaction targeting melanocytes. The most probable cause involves a complex interplay of immune cells and immuno-genetic factors. Specifically, abnormalities in T cell subsets and changes in melanocyte antigens may lead to heightened autoreactive CD8+ and CD4+ T cells, along with the production of autoantibodies against melanocytes. While the precise triggering factors for autoimmunity in vitiligo patients remain uncertain, physical trauma and oxidative stress on melanocytes are implicated as potential contributors.

Vitiligo, affecting 1% of the European and U.S. population, involves immune dysregulation triggered by oxidative stress in genetically predisposed individuals (Figure 4) [15]. Corticosteroids, immunomodulators, and phototherapy are conventional treatments, yet patient satisfaction varies [16]. Nutritional approaches, enhancing traditional therapies, have been explored. Studies highlight the roles of gluten, phenylalanine, zinc, fatty acids, and vitamins B12, C, E, and D in vitiligo [17]. Ginkgo biloba exhibits anti-inflammatory effects, slowing disease progression [18]. Curcumin, when topically applied with narrowband ultraviolet B (NB-UVB) phototherapy, promotes skin repigmentation [19]. Polypodium leucotomos demonstrates photoprotective effects [20]. Functional foods, such as capsaicin, carotenoids, and khellin, exhibit protective effects in combination with phototherapy [21]. While promising, further research is needed to uncover new therapeutic possibilities, emphasizing the importance of a balanced diet and proper integration in vitiligo treatment.

Atopic Dermatitis (AD)

**Figure 5:**
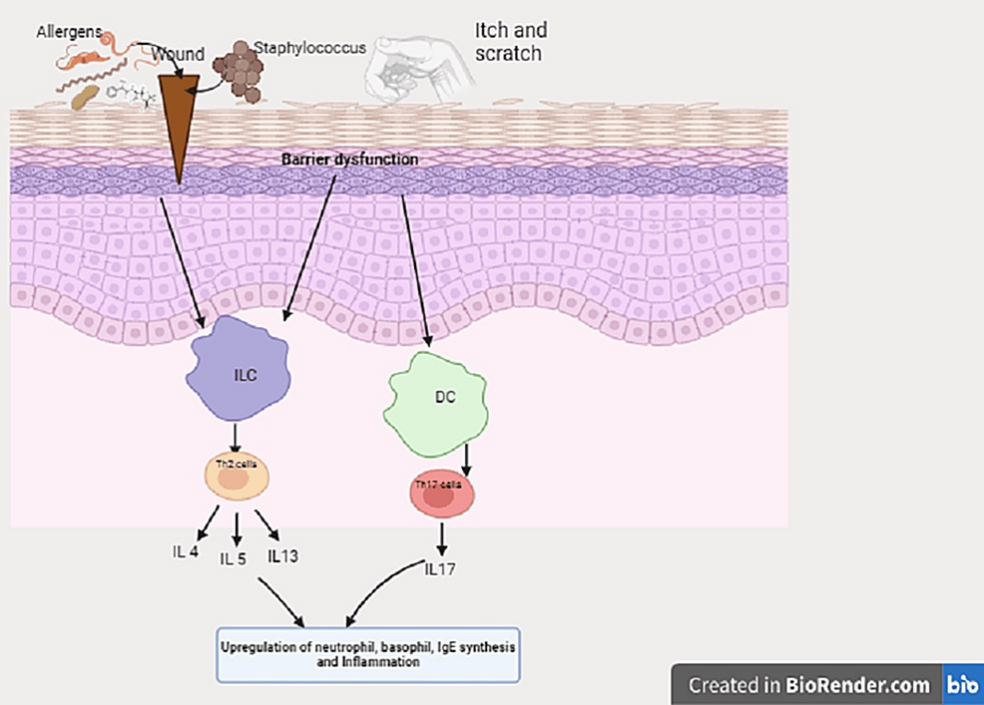
Pathogenesis of atopic dermatitis with skin barrier defects The impact of infections, allergens, and itch leads to upregulation of inflammatory pathways.

AD, affecting 5-20% of children and 1-3% of adults, is an inflammatory skin disorder linked to T2-mediated pathways and atopic comorbidities (Figure 5) [22]. Nutrition's role as a trigger remains debated. Individuals with AD were found to have dysbiosis of gut microbiota, which may alter the immunologic tolerance of mucosa, causing inflammation and affecting skin conditions. Dietary fiber and prebiotics consumption may have a role in reversing dysbiosis and may be helpful in AD. Maternal dietary restrictions during pregnancy or lactation show no impact on AD incidence or severity, while breastfeeding seems protective if the mother avoids certain foods [23]. The commonest foods children with AD are allergic to are eggs, milk, and peanuts. Therefore, unsupervised dietary restrictions, common in AD patients, may hinder growth without clear benefits [24]. Understanding immunoglobulin E (IgE) and non-IgE-mediated mechanisms connecting food intake to AD remains challenging. The foods that may raise IgE levels by triggering anaphylaxis are milk, egg, soy, wheat, peanuts, tree nuts, fish, and shellfish. Flavonoids lack substantial evidence, but ω-3 and ω-6 oils may alleviate symptoms. Vitamin A, C, D, and E supplementation, along with docosahexaenoic acid (DHA) and fish oil, demonstrates symptom improvement [23]. Vitamin D and probiotic's role is limited in AD and still debatable [25].

Dermatitis Herpetiformis (DH)

**Figure 6:**
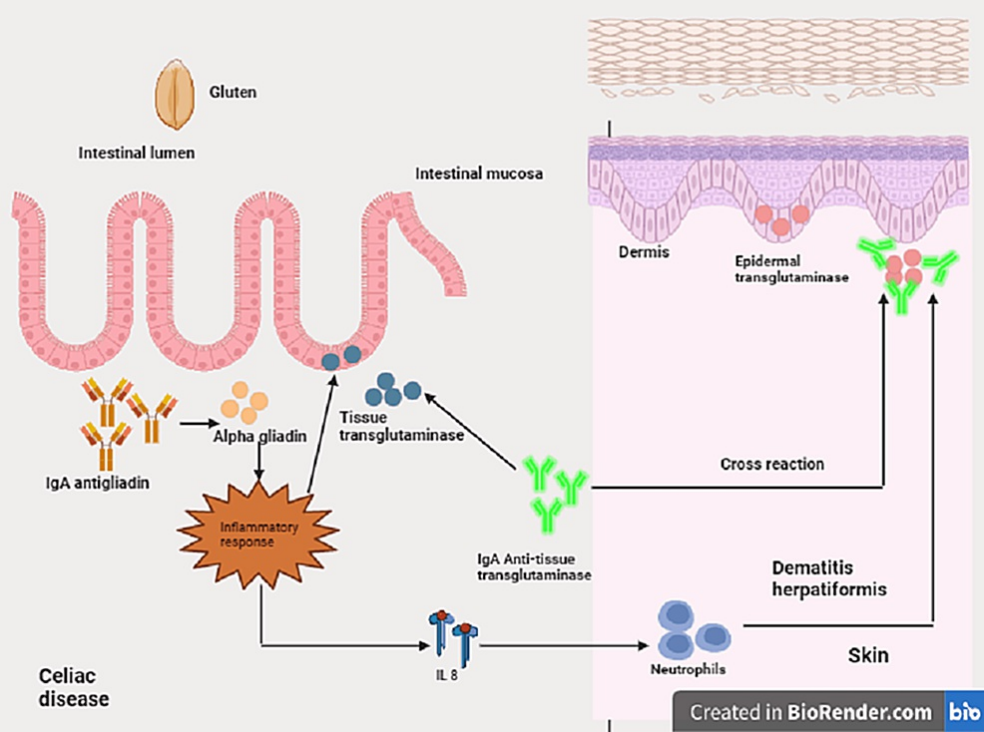
Pathogenesis of dermatitis herpetiformis The major environmental factor involved in triggering the disease is exposure to gluten. Gluten is composed of two peptides, gliadin and glutenin. The fraction linked to intestinal disease is from the gliadin, and its immunoreactivity is due to the N-terminal. In addition to antibodies directed precisely against gliadin in the intestinal mucosa, the formation of specific antibodies against autoantigens, such as transglutaminases, may also occur. Epidermal transglutaminase (ETG) is present in keratinocytes and maintains the stratum corneum's integrity. It performs its function by connecting the various epidermal structural proteins. Tissue transglutaminase (TTG) is the main antigen for CD antibodies, as ETG is the antigen in DH. Anti-TTG antibodies may, by cross-reaction, recognize ETG, leading to the onset of cutaneous IgA deposits.

DH, a cutaneous manifestation of celiac disease (CD), presents with granular IgA deposits and affects various body areas symmetrically [26]. Patients with DH have serum IgA antibodies against epidermal transglutaminase and tissue transglutaminase (Figure 6). Symptoms typically emerge at 30-40 years. The classic clinical presentation involves papules, vesicles, and urticarial plaques on limb extensor surfaces, buttocks, sacral region, neck, face, and scalp [27]. A lifelong GFD is the primary treatment, addressing both gastrointestinal and cutaneous manifestations. GFD's efficacy varies, taking three to six months for intestinal symptoms and up to one to two years for complete cutaneous resolution [28]. Despite its importance, patient adherence to GFD is challenging due to social, economic, and availability factors. Nutrition education is crucial, particularly for celiac children and teenagers, to enhance GFD compliance and prevent complications.

Chronic Spontaneous Urticaria

**Figure 7:**
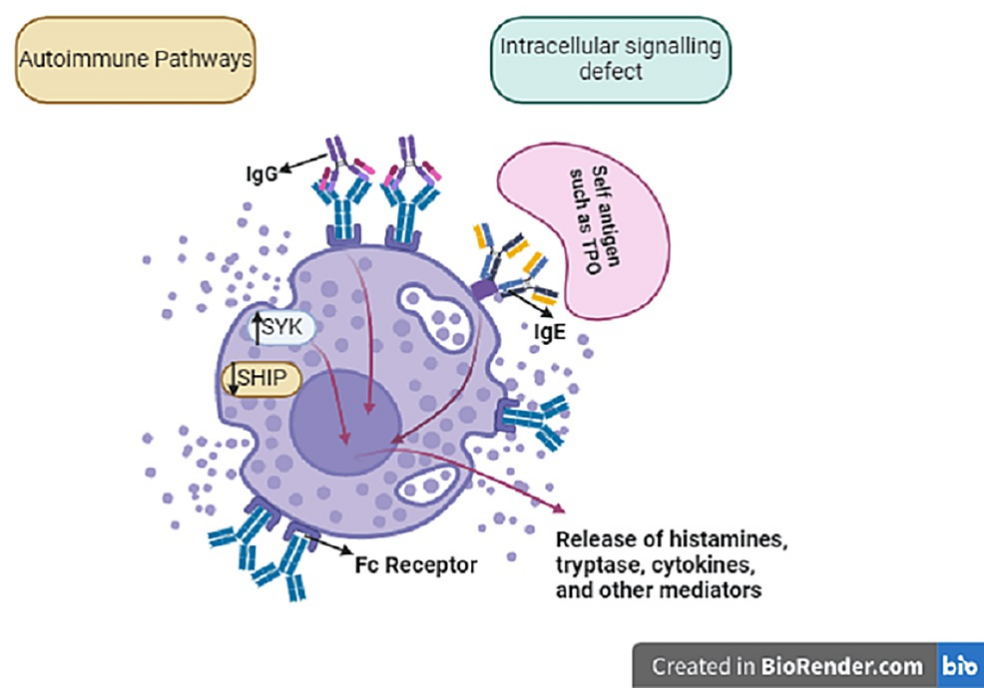
Pathogenesis of chronic spontaneous urticaria The abnormal activation of mast cells and basophils in patients with chronic spontaneous urticaria (CSU) is believed to result from two primary mechanisms: intracellular signaling defects and autoimmune processes. In the former, improper activation of molecules such as spleen tyrosine kinase (SYK) or the inhibition of negative regulators such as the Src homology (SH2)-containing inositol phosphatases (SHIP), leads to the spontaneous degranulation of mast cells/basophils, resulting in the release of histamine and other protein and lipid mediators. The prevailing theory regarding the pathogenesis of CSU involves antibody-mediated activation of mast cells and basophils, which can occur through IgG- or IgE-mediated pathways.

Chronic urticaria (CSU) is a prevalent inflammatory skin condition causing transient, pruritic wheals, affecting 9% of the global population and impacting their quality of life [29]. The exact trigger for mast cell activation remains unclear, with potential causes including immunologic and non-immunologic pathways (Figure 7) [30]. While CSU patients often consider dietary modifications, the etiopathogenic role of food is uncertain, leading to controversy over dietary strategies. Elimination diets lack strong evidence, with food additives and personalized diets recommended cautiously [31]. Allergenic substances may be eliminated in proven food allergies. Vitamin D and probiotics show promise as supplementary therapies [29]. Elimination diets should be monitored for at least three weeks, considering local dietary habits, to assess response without risking nutritional deficiencies or impaired quality of life. Bridging the knowledge gap on diet in CSU is crucial for informed treatment decisions.

Acne

**Figure 8:**
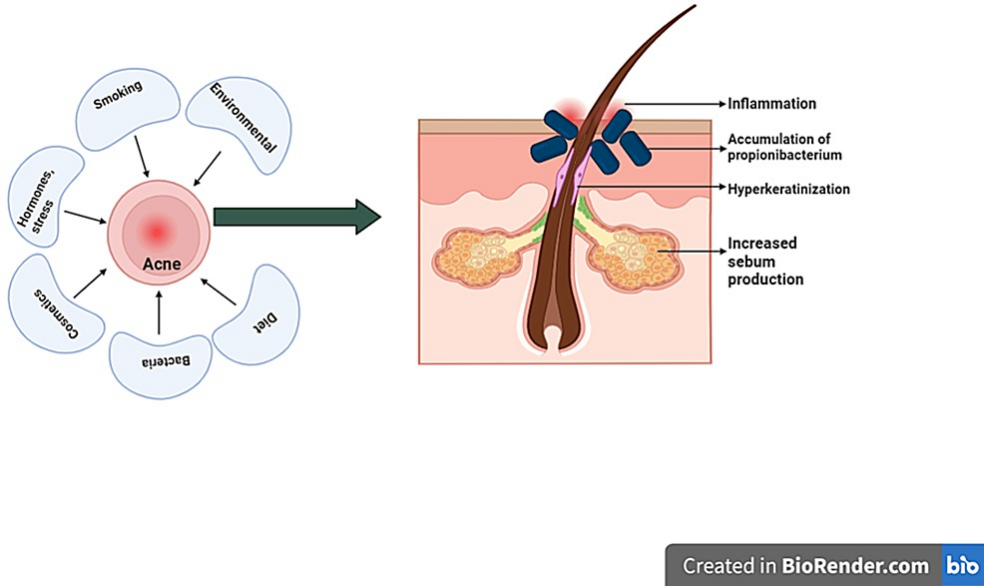
Acne pathogenesis Various factors associated with acne and the interplay of increased sebum production, accumulation of propionibacterium, hyperkeratinization, and inflammation, resulting in the formation of comedones.

Dermatologists historically dismissed a diet-acne link, but recent research highlights a significant connection. The health of the gut microbiota is thought to influence skin health and the development of acne through its effects on the immune system and the level of inflammation it is programmed to create (Figure 8). Studies reveal associations between acne and dairy consumption, particularly with skim milk due to its growth hormones and steroids [31]. The Western diet, high in glycemic load foods such as sugar and refined grains, has been implicated in acne pathogenesis. Rapid glucose absorption raises insulin and IGF-1 levels, which positively correlate with the severity of acne, stimulating sebum production and androgen synthesis. Randomized controlled trials support this, indicating that a 10-week low glycemic load diet and probiotics improve acne, enhancing insulin sensitivity and reducing androgen-related factors [32]. Additionally, polycystic ovarian syndrome (PCOS) exacerbates acne, as heightened androgen levels result from insulin excess [32]. Eliminating sugar and carbohydrates can normalize hormones, decrease oil production, and alleviate acne in PCOS individuals.

Scleroderma

**Figure 9:**
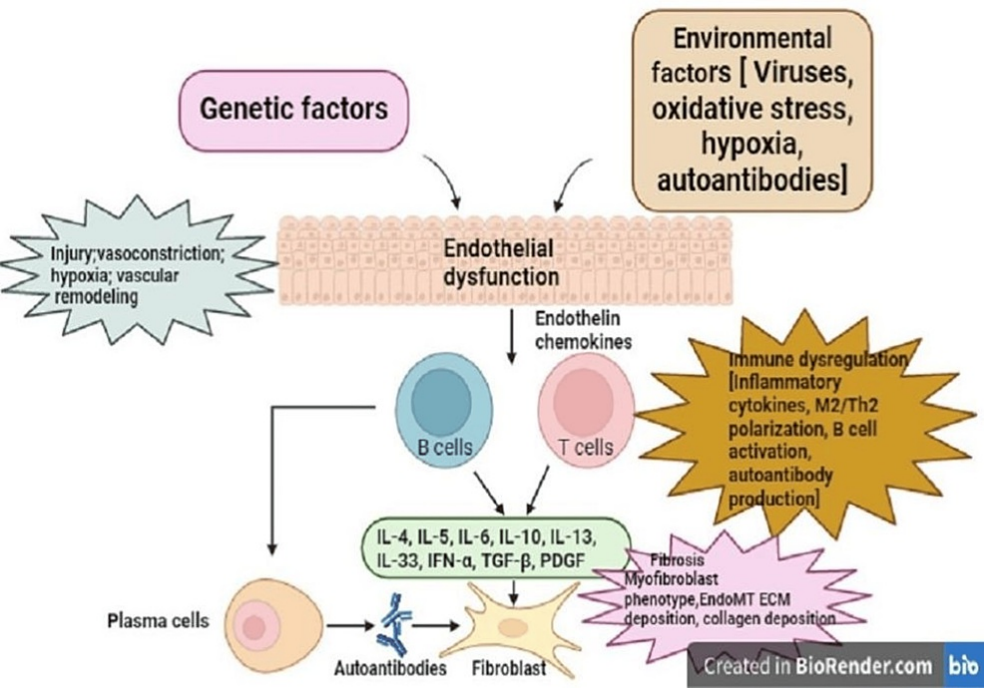
Pathogenesis of scleroderma The first site of persistent immune-mediated inflammation is most likely the microvessel wall, causing alterations involving not only the endothelium, but also all the vessel layers, with subsequent myofibroblast activation, excessive extracellular matrix (ECM) deposition, and unrestrained tissue fibrosis.

Systemic sclerosis, characterized by immune dysfunction and excessive collagen production, results in cutaneous sclerosis and organ fibrosis [33]. While no direct evidence links specific foods to reduced collagen formation, dietary choices can impact fatigue, inflammation, and digestive issues associated with the condition (Figure 9). Despite efforts to maintain a nutritious diet, individuals with scleroderma face an increased risk of malnutrition [34]. Challenges in mastication, deglutition, and meal preparation contribute to insufficient nutrient intake, leading to weight loss. Even those with adequate food consumption may struggle with nutrient absorption, risking deficiencies [35]. Monitoring nutritional status and adhering to a balanced diet is crucial. A notable weight reduction within three to six months may signal insufficient nutrient and calorie intake, emphasizing the need for regular weight monitoring in scleroderma patients [36].

Table 1 provides a list of food items that can have a positive or negative impact on a particular skin condition.

**Table 1:** Summary of the food elements that should be included or avoided in a diet in a particular skin condition

Disease	Gender & Age	Negative Foods	Positive Foods
Psoriasis	The overall estimated prevalence of psoriasis is similar between men and women. In women, psoriasis usually manifests at 16 to 22 years or 55 to 60 years. In men, these 2 peaks for age at onset occur more around 30 to 39 years and 60 to 79 years [5].	Gluten-rich foods and foods rich in vitamin A	Fish oil supplementation (rich in omega-3 PUFAs, fresh fruits and vegetables such as carrots and tomatoes as they are high in carotenoids, flavonoides and vitamin C.
Alopecia areata	Women are more likely to develop alopecia areata than men. More than 80% show signs of the disease before age 40, and 40% experience symptoms by age 20 [13].	Gluten-containing foods, dairy products, ultra-processed foods, fried foods, and food and drinks with added sugars.	Gluten-free diet high in raw vegetables, soy, and protein, whole grains, legumes, and fruits such as citrus, cherries, apples, berries and grapes.
Pemphigus	Pemphigus foliaceus typically impacts both men and women at similar rates, although in certain populations, it appears to affect women more frequently. While the usual age range for onset of symptoms is between 40 and 60 years old, in certain regions, manifestations of the disease may start during childhood [11].	Foods containing thiols, thiocyanates, phenols and taninssuch as garlic, cabbage, cauliflower, mustard, mango, banana, blackberry, coffee, tea, wine, beer, candy, and baked foods.	Fresh vegetables such as carrots and peas, cottage cheese, yogurt, fish, eggs, oatmeal, fruit and vegetable smoothies.
Vitiligo	Equal distribution in both genders and all ethnic groups. The disease can develop at any age. However, for many people with vitiligo, the white patches begin to appear before age 20 and can start in early childhood [16].	Butter, margarine, milk, milk products, red meat, desserts, sugar, soft drinks, potatoes.	Olive oil, corn, tomato, avocado, legumes, apples, onion, garlic, eggplant, berries, grapes.
Atopic dermatitis	AD is a prevalent condition among young adults, with a higher incidence in females than males by the age of 24. However, the onset of atopic dermatitis in adulthood appears to affect both sexes equally during young adulthood [22].	Wheat, milk, soy, fish, eggs, and peanuts.	Egg-free diet in infants, supplementation of probiotic intestinal bacteria.
Dermatitis herpetiformis	DH affects mostly adults and slightly more males than females. The mean age at onset is about 50 years [26].	Foodstuffs containing gluten such as wheat, rye, oats, and barley. Iodine-containing foods such as fish and iodized salt should be avoided. Additionally, vitamins should be avoided in patients who do not respond to a gluten-free diet.	Gluten-free diet, rice, corn, and potatoes.
Urticaria	Women are more affected by chronic urticaria. In most studies, the peak age of CU occurrence is between 20 and 40 years [29].	Foods with added ingredients such as salicylates, benzoates, and tetrazine. Avoid foods containing aromatic volatile ingredients such as herbs, tomatoes, and white wine.	Foods low in histamines such as most vegetables, fresh meat, fish like salmon, cod, and trout, dairy other than cheese and yogurt, bread, and rice.
Acne	Acne is more common in women than in men at all ages after age 20 years. Variations in the prevalence of the disease among different ethnic groups have also been reported [31].	Sugars, refined grains, fast food, chocolate, greasy food, whey protein powder, foods rich in Omega-6 Fats.	High-fiber foods such as oatmeal, beans, apples, carrots, salmon fish ( Omega-3 fatty acids) and nuts.
Scleroderma	It is more common in women than men. Most localized types of scleroderma show up before age 40, and systemic types of scleroderma are typically diagnosed between ages 30 and 50 [34].	Processed foods and foods with artificial ingredients, preservatives or hydrogenated oils. Foods containing sucrose, evaporated cane juice, fructose, brown rice syrup, molasses, corn syrup and lactose containing dairy products.	Whole wheat bread, cereals, barley, quinoa, oats, anti-inflammatory herbs and spices such as cinnamon, ginger paprika, turmeric, and curry leaves.

Dietary Recommendations

Table 2 outlines key dietary recommendations for optimizing the skin-gut axis, highlighting the crucial role that nutrition plays in maintaining skin health and gut microbiome balance. From early infancy to adulthood, dietary choices profoundly influence microbial populations in the gut, which in turn impact skin conditions and overall well-being [37-39]. From breastfeeding practices to dietary fat intake, protein-rich diets, fiber consumption, antibiotics, and probiotics, each aspect of nutrition plays a vital role in shaping the delicate balance between gut and skin health. Let us delve into these dietary recommendations to understand their implications for promoting optimal skin health and mitigating various skin conditions.

**Table 2:** Essential dietary guidelines to enhance the skin-gut axis, emphasizing the critical role of nutrition in preserving skin health and balancing the gut microbiome

Dietary Recommendation	Advantages/Disadvantages
Breast milk versus formula	Infants who are breastfed have substantially greater amounts of actinobacterial germs. Breastfeeding affects the variety of the gut microbiota by promoting the colonization of genera such as Lactobacillus and Bifidobacterium [40]. In contrast, infants who are fed formula frequently experience colonization of the stomach by the bacterial class -Proteobacteria, which contains several proinflammatory species [41]. Additionally, the opportunists Escherichia coli and Clostridium difficile as well as members of the phylum Bacteroides are more likely to invade infants who are fed formula [42]. Levels of oligosaccharides and different fatty acids in breast milk have a good impact on the gut microbiome and its metabolites, which help fight allergies and asthma by activating Tregs [43].
Diets high and low in fat	High trans-fat diets may lead to inflammation and skin irritation [44]. alcohol and high-fat diets can exacerbate skin inflammation and slow wound healing [45]. Low-fat diets may reduce gut bacteria diversity and increase systemic inflammation.
Protein-rich diet	The skin can be protected from aging and can speed up the healing of wounds with a high-collagen peptide diet, which contains large amounts of microorganisms [46]. When whey and pea protein extracts are included in the diet, the prevalence of pathogenic Bacteroides fragilis and Clostridium perfringens decreases while the abundance of gut-commensal taxa, such as Lactobacillus and Bifidobacterium, increases [47]. SCFA levels, which are vital for maintaining the mucosal barrier, are raised in the intestinal mucosa when pea proteins are consumed [48].
Fiber-rich diet	Consuming whole grains, particularly those rich in dietary fiber, increases the populations of Bifidobacteria and Lactobacillus/Enterococcus [49]. Foods containing complex carbohydrates can be converted into soluble fatty acids (SCFAs) through fermentation by the gut microbiome [50]. SCFAs enhance mucosal immunity, modulate respiratory diseases, prevent inflammatory disorders, regulate lipid and glucose metabolism, and inhibit metabolic by-products [51]. SCFAs also play a role in Foxp3 (Forkhead box protein P3) expression, regulating Treg function and enhancing regulatory T cell function. SCFAs can, in addition, influence skin microbial groups, affecting immune defense mechanisms and promoting skin homeostasis.
Antibiotics	By eliminating or preventing the growth of particular microbial groups and altering the molecular patterns linked to the microbiome, antibiotics have the potential to alter the makeup and functionality of the gut microbiome [52]. When used orally to treat skin wounds, vancomycin, for instance, decreased bacterial diversity and the expression of the regeneration gene III gamma (RegIII), potentially slowing the healing process [53].
Probiotics	Probiotics can prevent gut colonization by pathogens and support anti-inflammatory responses [54]. Common probiotic microbes include Bacillus, Bifidobacterium, Enterococcus, Escherichia, Lactobacillus, Saccharomyces, and Streptococcus [55]. Probiotic consumption has been shown to improve skin sensitivity, restore barrier function, and prevent dermatological diseases. Studies have shown that probiotics can improve skin health in patients with acne vulgaris, rosacea, and atopic dermatitis [56].

## Conclusions

While nutritional studies provide valuable insights into the relationship between diet and skin health, it is essential to recognize their limitations. Individual responses to diets and nutrients vary significantly, and confounding variables often complicate research outcomes. Despite these challenges, emphasizing overall eating habits over specific meals or nutrients appears beneficial for managing various skin conditions. Diets rich in whole foods, antioxidants, fiber, and phytonutrients show promise in promoting skin health and preventing related comorbidities. Incorporating prebiotics through whole foods diets may also help maintain a balanced gut microbiome, potentially benefiting inflammatory skin conditions. Furthermore, exploring specific food triggers and considering elimination diets under medical guidance can be valuable for managing certain skin conditions. While dietary supplements may have limited efficacy, personalized dietary approaches tailored to individual needs and preferences can be facilitated by integrative healthcare professionals.
